# Obituary—D. C. Ambler, M. D.

**Published:** 1866-07

**Authors:** 


### OBITUARY.

DROWNED, in the St. John's river, Florida, on the 4th of April, D. C. AMBLER, M. D., dentist.

It is a sad duty to record the sudden death of a so highly respected and beloved member of the profession. Dr. Ambler practiced, for many years, in the city of New York; but, for the last few years has resided in Florida. His circle of acquaintances was large; and men of education, refinement, and worth, were numbered among his most intimate friends. His enemies were few, (and no great and useful men are without them), but they were chiefly, if not solely, of his own profession, envious of his reputation and success.

He was a favorite pupil of the venerable Dr. Valentine Mott, and graduated at the College of Physicians and Surgeons, of the city of New York. His love of mechanics and chemistry, aided by an inventive and fertile mind, enabled him to make rapid advances in dental surgery, which he chose as a specialty. He was awarded, in the year 1833, by the American Institute, a gold medal for artificial mineral, or porcelain teeth, the first awarded, it is thought, for any manufacture. He thus gave an impetus to this important and growing branch of dentistry, the growth of which has been so rapid, that it has become a specialty, giving employment, daily, to hundreds of busy hands. Sickness in his family called him away from this field of usefulness, to another in the Sunny South; leaving to others the laurels and riches which he truly merited.

I had the pleasure of meeting him in New York, last Fall, when on a visit from Jeffersonville. On that occasion he entertained me with an account of the many difficulties encountered by the early plodders in the field of dentistry, giving a history of his travels through the South, with a few incidents in the practice in this country, of the now famed Dr. Brewster, with whom he was intimate. I little thought, then, that it would be the last time that I should look upon his pleasant and genial face—the last time that I should listen to his cheerful and instructive conversation. Truly "in the midst of life we are in death." He was a sincere christian, a member and communicant of the Protestant Episcopal Church; and those left behind mourn not for him as those without hope—for, having finished his course in faith, he now rests from his labors. A. T.

At a meeting of the *Society of Dental Surgeons, of the city of New York,* held at their room, No. 24 Cooper Union, on the 25th of April, 1866, the death of D. C. Ambler, M. D., was announced to the meeting, when, on motion, a committee of three was appointed by the chair, to draft suitable resolutions on the exemplary character, and exalted professional worth of the deceased.

The unexpected close of an eventful life, in the career of enterprise and usefulness, cannot fail to arrest the attention of the most thoughtless, and shroud an appreciative community in the deepest gloom. Such was signally the case when the startling intelligence of the sudden death of Dr. D. C. Ambler, by drowning, on the 4th of April, in the St. John's river, Florida, reached us. In Dr. Ambler, we recognized an old familiar friend and professional brother, whom we all delighted to honor while living, and now sincerely mourn his death. Dr. Ambler was one of the Pioneers in the profession of dentistry, one who labored hard to elevate the standard of professional excellence; and the science and art of dentistry were materially advanced by his scientific knowledge and ingenuity; and to his experimental researches is our profession indebted for those improvements in mineral teeth, the manufacture of which has been carried on so extensively and with scch perfection in this country, therefore be it

*Resolved,* That this society show its affection for his many virtues, and appreciation of the bright example of our departed friend and brother, by placing on record these expressions of our bereavement, and sorrow for his departed worth.

*Resolved*, That our sympathies, true and heartfelt, are hereby tendered to the relatives and friends of the deceased, in this sad and inscrutable dispensation of Providence.

*Resolved*, That Dr. John Gardner Ambler, one of our members, and nephew of the deceased, be requested to address the profession, at such time and place as may be convenient to himself, in an *obituary* or *eulogy* of the deceased.

*Resolved*, That these proceedings be published in one or more daily papers of this city, and in the Dental Journals.

All of which is respectfully submitted.

T. H. BURRAS,
JOHN ALLEN,
CHAUNCEY P. FITCH,
Committee.

